# The HBx oncoprotein of hepatitis B virus potentiates cell transformation by inducing c-Myc-dependent expression of the RNA polymerase I transcription factor UBF

**DOI:** 10.1186/s12985-015-0293-5

**Published:** 2015-04-14

**Authors:** Pallavi Rajput, Surendra Kumar Shukla, Vijay Kumar

**Affiliations:** Virology Group, International Centre for Genetic engineering and Biotechnology, Aruna Asaf Ali Marg, New Delhi, 110067 India

**Keywords:** HBx, Hepatitis B virus, Upstream Binding Factor, rDNA transcription, c-Myc, Cell proliferation, Cell transformation

## Abstract

**Background:**

The HBx oncoprotein of hepatitis B virus has been implicated in the development and progression of hepatocellular carcinoma (HCC). HBx engages multiple signalling and growth-promoting pathways to induce cell proliferation and enhance ribosome biogenesis. Interestingly, the levels of Upstream Binding Factor (UBF) required for rDNA transcription and ribosome biogenesis are found elevated in the HCC patients. However, the molecular mechanism of UBF overexpression under the HBx microenvironment and consequent cell transformation remains elusive.

**Methods:**

The UBF gene expression was investigated after co-expressing HBx in immortalized human hepatocytes (IHH) and human hepatoma Huh7 cells. Gene expression analysis involved estimation of mRNA level by real-time PCR, western blotting of protein, chromatin immune-precipitation assay, BrdU incorporation assay and soft agar colony formation assay. UBF expression was also investigated in an HBx transgenic mouse model of HCC to get a better mechanistic insight under more physiological conditions.

**Results:**

Ectopic expression of HBx in IHH as well as Huh7 cells led to a marked increase in UBF expression both at mRNA and protein levels. Elevated levels of UBF were also observed in the hepatic tumors of HBx transgenic mice. Our ChIP studies revealed a marked increase in the occupancy of c-Myc on the UBF gene promoter in the presence of HBx and increase in its transcription. Enhanced UBF expression under the HBx microenvironment led to a marked increase in cell proliferation and transformation of IHH cells.

**Conclusions:**

Our study provides some compelling evidences in support of HBx-mediated increase in UBF levels that abets oncogenic onslaught in hepatic cells by increasing rDNA transcription and ribosome biogenesis.

**Electronic supplementary material:**

The online version of this article (doi:10.1186/s12985-015-0293-5) contains supplementary material, which is available to authorized users.

## Background

Hepatocellular carcinoma (HCC) is the one of the most prevalent human cancer causing third largest cancer related deaths worldwide. Chronic Hepatitis B virus (HBV) infection contributes to more than half of the observed liver cancer cases and thus is a major risk factor of HCC [1]. The HBx oncoprotein encoded by the X gene of HBV is the main viral oncoprotein involved in development of HCC; although molecular mechanism of HBx-mediated HCC is still not fully understood [2]. HBx has been shown to activate several growth signalling pathways and gene promoters, albeit it does not directly interact with DNA. The transactivation function of HBx has been extensively reviewed in the context of cell cycle, cell growth and proliferation that is significantly altered during HCC [3-6]. HBx modulates the expression profiles of host genes by engaging certain transcription factors. The HBx-responsive genes typically carry binding sites for c-Myc, Nuclear factor–kB (NF-κB), Activator protein-1 (AP-1), CCAAT/enhancer binding protein (CEBP), Activating transcription factors/ c-CAMP response element binding protein ATF/CREB, or the calcium-activated factor NF-AT, apparently stimulating their binding to the promoter elements [7]. Apart from Pol II-dependent promoters, HBx can modulate the RNA Polymerase I activity which is characteristic of several neoplastic growth [5]. However, the molecular mechanism of HBx-mediated RNA Polymerase I activity regulation remains elusive.

The Upstream Binding Factor (UBF) is considered as a major transcriptional regulator of RNA Pol I dependent rRNA genes. UBF acts predominantly in the promoter region and facilitate the loading of SL1 and RNA pol I. Therefore, efficient binding of UBF to rRNA gene is a pre-requisite for formation and assembly of initiation complex [8-10]. In mammalian cells, the UBF gene expression is regulated at both transcriptional and post-transcriptional levels. During development and differentiation, a diminished expression of UBF positively correlates with decreased rRNA synthesis while the cell cycle-dependent rRNA synthesis is primarily regulated through post-translational modifications of UBF [8,11]. Not surprisingly, the intracellular level of UBF is a key determinant of active rRNA genes [11]. Despite a critical role played by UBF in ribosome biogenesis, nothing much is known about the regulation of UBF gene expression except for the involvement of some growth regulators such as EGF, IGF and c-Myc in the process [12-16].

Taking into consideration, the complex interplay between ribosome biogenesis and neoplastic transformations, it is not surprising to observe the altered levels of UBF in many cancers such as breast and hepatic cancers [17,18]. Importantly, elevated levels of UBF have also been reported in some HCC patients [18]. Since the HBx oncoprotein of HBV is the major causative agent in the development of HCC, we wondered if UBF expression and activity was essential for hepatocarcinogenesis.

In the present study, we show that HBx can stimulate the expression of UBF gene with the help of transcription factor c-Myc. Further, UBF expression was essential for enhanced cell proliferation and transformation under the HBx microenvironment.

## Results

### HBx induces the expression of UBF in hepatic cell lines

**Figure 1:**
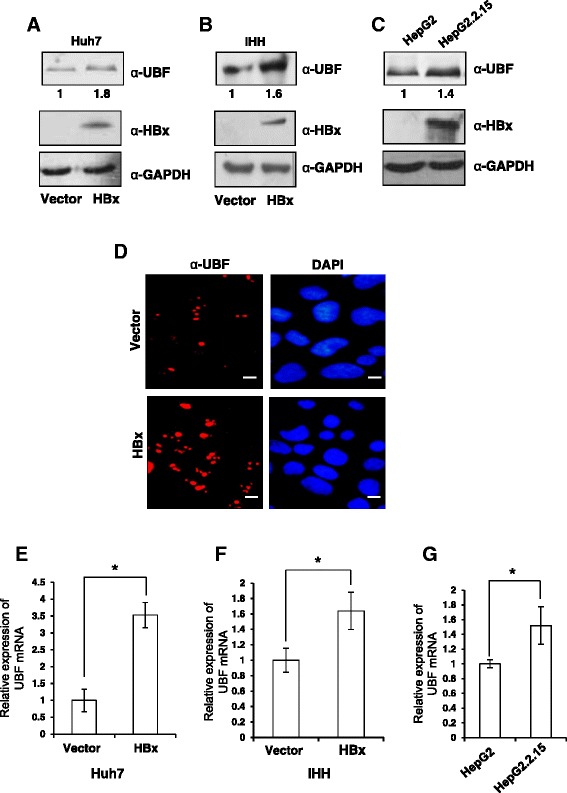
Regulation of UBF expression under the HBx microenvironment. **(A) & (B)** Huh7 and IHH cells were transiently transfected with vector or HBx expression plasmids. After 48 h, cell lysates were western blotted for UBF, HBx and GAPDH. **(C)** HepG2 and HepG2.2.15 cell extracts were western blotted for UBF, HBx and GAPDH as above. **(D)** Huh7 cells were transiently transfected as in panel A and processed for immunofluorescence with anti-UBF antibody (Red). DAPI staining (Blue) was used to visualize nuclei. Scale bar represents 50 μm **(E) & (F)**, Huh7 and IHH cells were transiently transfected as in panel A, B and total RNA was isolated for measuring UBF mRNA levels by RT-qPCR using primers mentioned in Additional file 1: Table S1 **(G)** The UBF mRNA levels in asynchronously growing HepG2 and HepG2.2.15 cells was measured as above. All data are represented as mean ± S.D of three independent experiments. * represents a statistically significant difference of p <0.05.

Deregulation of UBF levels is frequently seen in cases of HCC [18]. Therefore, the role of HBx in development of HCC was investigated under HBx microenvironment. The role of HBx in UBF expression was investigated after transiently transfecting Huh7 and IHH cells with either vector or HBx expression plasmid and monitoring the UBF protein level by western blotting. There was a marked increase in UBF expression in the HBx transfected cells as compared to control transfected cells (Figure 1A and B). As expected, elevated levels of UBF were also observed in HepG2.2.15 cells that carry an integrated copy of HBV genome and express HBx in comparison to the parent HepG2 cells (Figure 1C). Further, our immunofluorescence studies confirmed increased UBF expression in the presence of HBx (Figure 1D). As HBx is implicated in the transcriptional up-regulation of many cellular genes, we next investigated the transcriptional regulation of UBF gene by HBx. RT-qPCR analysis of the RNA isolated from the HBx-expressing cells confirmed a highly significant increase (p < 0.05) in the UBF transcripts (Figure 1E, F and G). Collectively, these results indicate that viral HBx can induce the expression of UBF required for RNA polymerase I activity.

### Elevated levels of UBF is found in the hepatic tumours of X15-*myc* transgenic mice

**Figure 2:**
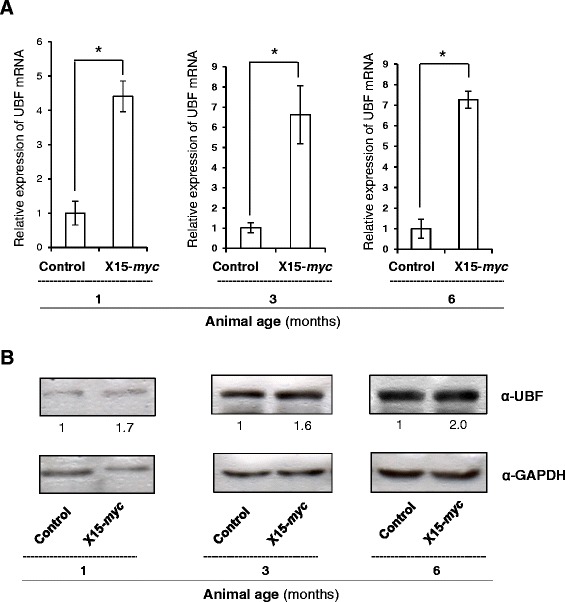
Expression levels of UBF in the hepatic tumours of X15- *myc* transgenic mice. **(A)** Total RNA from the liver tissues of 1, 3 and 6 months old control and transgenic mice was isolated and subjected to RT- qPCR as described above. **(B)** Total cell lysate of liver tissues of 1, 3 and 6 months old control and transgenic mice were western blotted for UBF, HBx and GAPDH. Data are shown as mean ± S.D of three independent experiments. * represents a statistically significant difference of p <0.05.

As we observed elevated expressions of UBF in the presence of HBx, we next investigated UBF expression in a tumour environment [19]. Analysis of the X15-*myc* transgenic mice (different stage hepatic tumours) revealed a significant increase in UBF expression both at mRNA (p < 0.05) as well as protein levels in comparison to control mice (Figure 2A and B). Further, the immunohistochemical analysis confirmed elevated UBF expression in the transgenic liver tissues (see Additional file 1). These observations confirmed an *in vivo* up-regulation of UBF under the HBx microenvironment.

### HBx modulates UBF expression by engaging c-Myc on the UBF promoter

**Figure 3:**
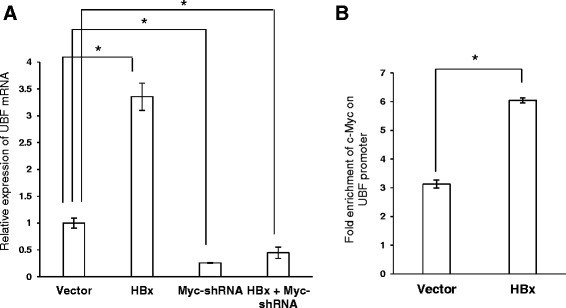
c-Myc- dependent transcriptional regulation of UBF in the presence of HBx. **(A)** Huh7 cells were transiently transfected with vector, HBx and/or Myc- shRNA plasmids. Total RNA was isolated and the levels of UBF mRNA were measured by RT-qPCR. **(B)** IHH cells were transiently transfected with vector or HBx expression plasmids. After 48 h, cells were subjected to ChIP assay using 'anti-Myc antibody. Pre-immune sera were used as a negative control. Fold DNA enrichment over mock due to UBF occupancy was measured by ChIP-qPCR using primers specific for UBF promoter (Additional file 1: Table S1). Data are shown as mean ± S.D of three independent experiments. * represents a statistically significant difference of p <0.05.

HBx is known to regulate the levels of many host proteins either by engaging different transcription factors or by interfering with their intracellular stability [7]. To understand the molecular mechanism of UBF gene expression in the presence of HBx, we first examined the role of transcription factors such as c-Myc which is already reported to act as a key activator of UBF gene expression [13]. Since viral HBx is known to stabilize c-Myc and exhibit an oncogenic cooperation with it, we next probed the regulation of UBF via c-Myc [20,21]. In accordance with the stimulatory role of c-Myc on UBF promoter, RNA interference against c-Myc using specific shRNA, not only led to a dramatic decrease in the UBF gene expression (p < 0.05) but also prevented the HBx-mediated gene stimulation (p < 0.05) (Figure 3A). Thus, c-Myc appeared to be indispensable for UBF gene stimulation under these conditions. The regulatory action of c-Myc on UBF was further investigated for its promoter occupancy in the presence of HBx [13]. Our ChIP studies confirmed a surge in the recruitment of c-Myc on the UBF promoter in the presence of HBx (p < 0.05) (Figure 3B). Based on these observations, we conclude that viral HBx stimulates UBF gene expression by enhancing the promoter recruitment of transcriptional activator c-Myc.

### UBF cooperates with HBx to stimulate cell proliferation and tumorigenesis

**Figure 4:**
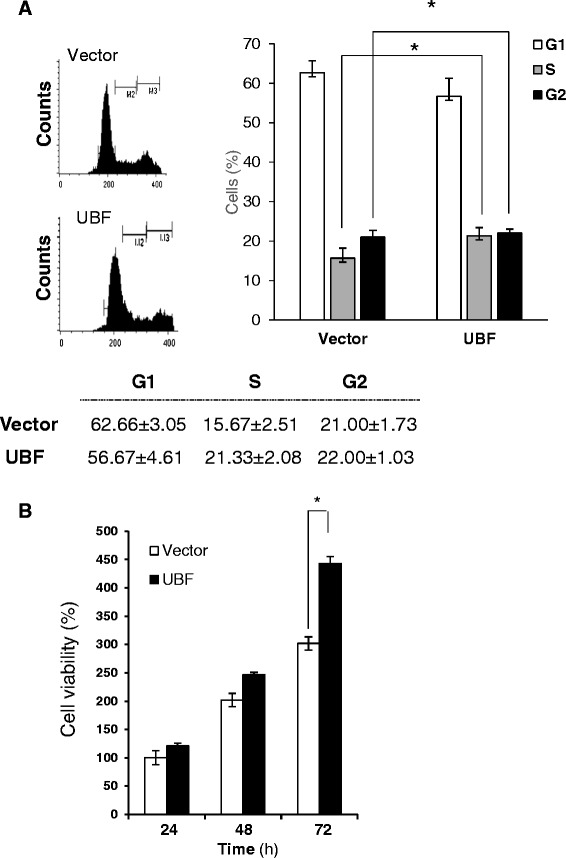
Effect of UBF overexpression on cell proliferation and cell survival of hepatoma cells. **(A)** Cell cycle distribution of vector or UBF transfected Huh7 cells represented as percentage of total cells in different phases. **(B)** Huh7 cells were transiently transfected with vector or UBF expression plasmids. Cell viability was measured by MTT assay. All quantitative values are mean ± S.D of three independent experiments. * represents a statistically significant difference of p <0.05.

**Figure 5:**
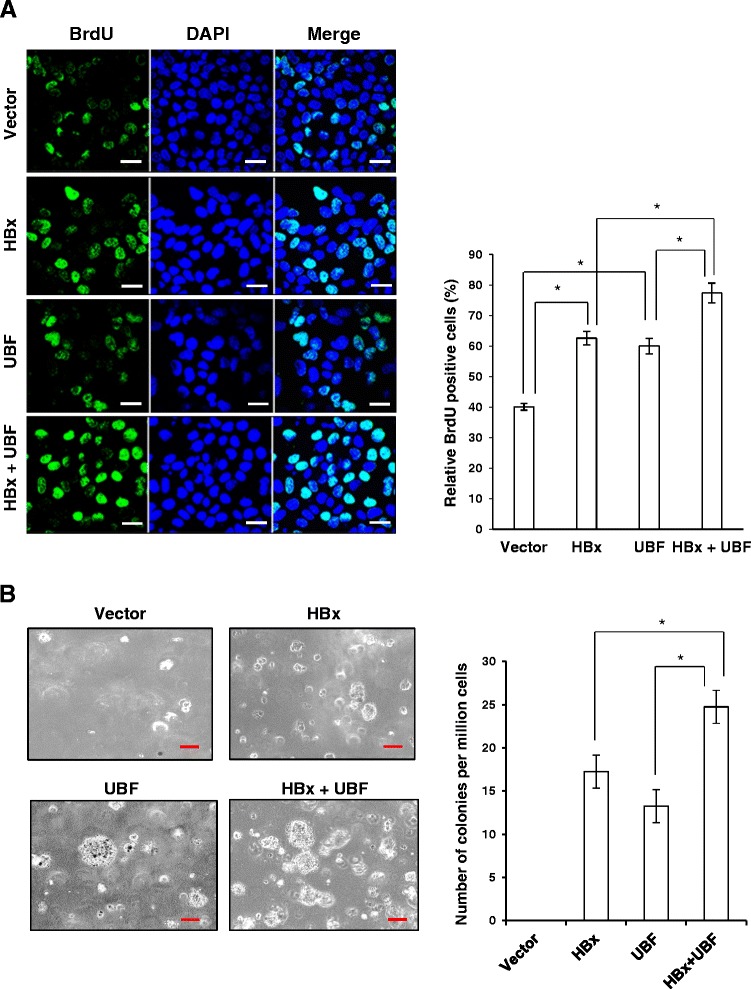
Cooperation between UBF and HBx in cell proliferation and transformation. **(A)** Huh7 cells transiently transfected with indicated plasmids are synchronized in S-phase by thymidine block. Following BrdU incorporation, cells were subjected to staining with antibody against BrdU and counterstained with DAPI, and the number of BrdU-positive cells was counted. Scale bar represents 50 μm. **(B)** IHH cells were transfected with indicated plasmids and allowed to grow in soft agar for 15 days and visualized under microscope for colony formation. Scale bar represents 10 μm. All quantitative values are mean ± S.D of three independent experiments. * represents a statistically significant difference of p <0.05.

UBF has been recognized as a critical regulator of rRNA synthesis which constitutes nearly 80 per cent of the total RNA pool and thus, a major component of the ribosome - the cellular machinery for protein biosynthesis [8]. Since HBx plays an extensive role in cell cycle progression and cellular proliferation, we wondered if enhanced expression of UBF under the HBx microenvironment also contributed towards these processes. Keeping these issues in mind, we first studied the effect of UBF over-expression on proliferation and survival of hepatic cells. As shown in Figure 4A, we observed a significantly (p < 0.05) accelerated entry of cells into S phase following UBF overexpression. Consistently, after 72 hours post-transfection UBF overexpressing hepatic cells exhibited significantly (p < 0.05) better survival in comparison to control cells. (Figure 4B). The observation was further characterized in presence of HBx by BrdU incorporation cell proliferation assay following ectopic expression of UBF and HBx. There was a significant increase (p < 0.05) in the proliferation of cells expressing either HBx or UBF alone. However, the cells co-expressing HBx and UBF showed significantly higher (p < 0.05) BrdU incorporation in comparison to cells expressing HBx or UBF alone (Figure 5A). These results suggested that HBx and UBF act cooperatively to drive cell proliferation in hepatic cells.

In line with the preceding observations, we further investigated the cooperative oncogenic capacity of HBx and UBF using a soft agar cell transformation assay. IHH cells transfected with UBF and HBx were examined for colony formation under phase contrast light microscope (Figure 5B). As anticipated, cells co-expressing UBF and HBx showed increased average size and number of colonies formed in comparison to HBx or UBF alone. Together, these results suggested a cooperative role of UBF and HBx in oncogenic transformation of hepatocytes.

## Discussion

The transcriptional activity of RNA polymerase I is essential for ribosome biogenesis which is required for supporting the translational capacity of cells as well as cell proliferation. In this process, the upstream binding factor or UBF is engaged as a key transcriptional regulator [8-10]. Thus, the cellular levels of UBF determine the number of active rRNA genes and the rate of ribosome biogenesis [11]. Importantly, it has been previously reported that UBF is significantly up regulated in HCC patients [18]. In accordance to this, the NS5A protein of Hepatitis C virus has been reported to activate RNA polymerase I transcription through UBF phosphorylation [22]. On the other hand, the Hepatitis B virus oncoprotein HBx is also known for its pro-proliferative effects on hepatic cells through modulation of various cellular pathways [7]. Although, HBx has been implicated in the regulation of RNA polymerase I transcription via activated Rat Sarcoma (Ras) and TATA binding protein (TBP), there is no direct evidence associating viral HBx with UBF functions [5]. Therefore, keeping the above background into consideration, in the current study, we have probed the molecular mechanism of oncogenic cooperation between viral HBx and cellular UBF.

Considering a formidable trans-activation function associated with HBx, here we studied its role in the expression of UBF gene. Our results clearly suggested that HBx could induce UBF expression in different hepatic cell lines (Figure 1). Elucidating the molecular mechanism of HBx action, we show that UBF gene was a transcriptional target of HBx where the transcription factor c-Myc played a pivotal role in UBF expression. This is in perfect agreement with our previous observation where we have showed increased intracellular stabilization of c-Myc under the HBx microenvironment [21]. We believe that accumulated intracellular c-Myc in presence of HBx is associated with enhanced UBF gene expression and its pathological consequences. Not surprisingly, c-Myc accumulation was associated with a surge in its recruitment to the UBF gene promoter (Figure 3). These observations are in concord with the finding that proto-oncogene c-Myc mediates its oncogenic effects through deregulating the expression of its target genes [23]. The physiological relevance of c-Myc dependent UBF expression in the presence of HBx was also evident from its elevated levels in the hepatic tumour microenvironment of X15-*myc* transgenic mice (Figure 2). The histopathological changes in the X15-*myc* transgenic mice become evident in as early as 1 month old transgenic mice with concomitant expression of c-Myc resulting in the emergence of a multi-focal, well differentiated HCC by the end of 6 months [19]. Our current observation that UBF levels were significantly altered in even 1 month old transgenic mice with gradual increase in the 6 month old transgenic mice reinforced the importance of c-Myc dependent UBF expression in cell proliferation and transformation leading to HCC development.

Since UBF is a major transcription factor involved in rRNA gene transcription and ribosome biogenesis, it was of great relevance to study the patho-physiological consequence of increased UBF levels under the HBx microenvironment. Our studies indicate that surplus UBF was actively recruited to the rDNA in the presence of HBx, facilitating epigenetic alterations and increased rRNA levels (Ahuja et al., unpublished data) leading to enhanced ribosome biogenesis. UBF has also been implicated in the proliferation and differentiation of murine myeloid cells [24]. Now, we show that ectopic expression of UBF in hepatic cells was associated with accelerated G1-S transition and cell proliferation (Figure 4). This prompted us to investigate if there was cooperation between UBF and HBx that resulted in an increased cell proliferation and oncogenic transformation. Using BrdU incorporation and soft agar colony formation assay, we demonstrate that UBF and HBx cooperated during hepatic cell proliferation leading to their oncogenic transformation (Figure 5). Thus, it may be inferred that HBx-mediated oncogenesis can, in part, be owed to UBF being a regulator of RNA polymerase I transcription, resulting in increased cell proliferation and growth, ultimately leading to HCC.

Collectively, UBF appears to be an important mediator of the HBx oncogenic activity by stimulating ribosome biogenesis and contributing towards cell proliferation and transformation.

## Conclusions

**Figure 6:**
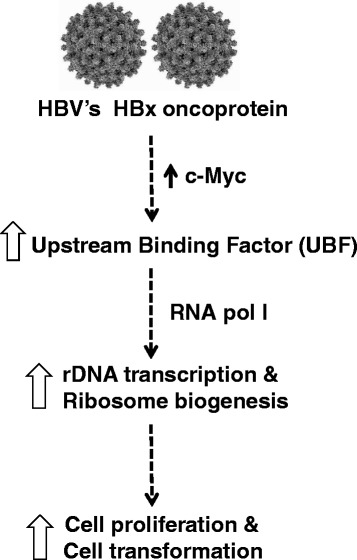
Schematic illustration depicting the mechanism of increase in UBF expression under the HBx microenvironment and its implication in development of HCC.

In conclusion, we show that Hepatitis B virus oncoprotein HBx induces c-Myc dependent transcription of UBF gene resulting in its enhanced expression in hepatic cell lines. As summarized in Figure 6, increased UBF expression cooperates with HBx to stimulate cell proliferation and oncogenesis.

## Methods

### Expression vectors and chemical reagents

The HBx expression plasmid has been described previously [19]. UBF-pcDNA expression vector was kind gift from Prof. Ross Hanahann, Peter Maculum Cancer Centre, Melbourne, Australia. Antibodies for HBx, UBF and c-Myc were purchased from Santa Cruz Biotechnology, USA. The Myc shRNA used in the study has been developed in our laboratory by cloning individual oligos directed against the transactivation and leucine zipper domain of c-Myc protein in pSilencerU6 1.0 vector [25].

Chemical reagents such as Propidium iodide and MTT were purchased from Sigma Chemical Co. (St Louis, MO). Dulbecco’s modified Eagle’s medium (DMEM), Fetal bovine serum (FBS), Streptomycin and Penicillin were purchased from Gibco BRL.

### Cell culture and transfection

The Immortalized Human Hepatocytes (IHH) cells were provided as kind gift from Dr. Fanny Daniel, Institut National de la Santé et de la Recherche Médicale Unite 481, Universite Paris 7, Paris, France. The maintenance of human hepatoma Huh7, HepG2, HepG2.2.15 cells have been described previously [26]. All cell lines were cultured in DMEM supplemented with 10% FBS and incubated at 37°C in a humidified chamber with 5-10% CO_2._ Seeding was done at a density of 0.6 million cells per 60 mm dish or 0.1 million cells/well (12-well plate) and transfection of all plasmid DNA was performed using Lipofectamine (Invitrogen, CA, USA) as per manufacturer’s protocol. Transfection efficiency was found ~70 per cent.

### Western blotting

Western blotting of protein samples was done as described previously [26]. Briefly, cells were directly harvested in 2X Laemilli’s buffer and boiled for 5 minutes in a water bath. The liver tissue samples were homogenized in the lysis buffer (20 mM Tris- HCl pH 7.5, 150 mM NaCl, 0.1% Triton X-100, 10% glycerol, 10 mM DTT, 1 mM Sodium Fluoride, 10 mM β -glycerol phosphate, 1 mM EGTA, 2 mM PMSF, 1.5 mM MgCl_2_) for 2 h at 25°C. The protein estimation was done by the Bradford assay. Equal amount of protein was loaded onto SDS gel and processed for western blotting. The analysis was done using Enhanced chemi-luminescence technique detected on a Kodak X-Ray film. The protein levels were quantified through densitometry using the ImageJ software.

### RNA isolation and real time quantitative PCR (RT-qPCR)

TRIzol reagent was used to isolate RNA as per manufacturer’s instructions. M-MuLV Reverse transcriptase (Fermentas) was used to reverse transcribe total RNA using oligo dT primers according to manufacturer’s guidelines. RT-qPCR was performed using Universal SYBR green mix (Biorad). ARPP mRNA was used as an internal control and the results were analyzed using comparative ΔΔCt method [27]. Primer sequences used are listed in Table S1 (see Additional file 1).

### Chromatin Immunoprecipitation assay (ChIP)

Chromatin immuno-precipitation assay was carried out according to manufacturer’s instructions (Upstate Biotechnology). Briefly, the crosslinked chromatin was immunoprecipitated using c-Myc antibody (N-262X, Santa Cruz). Subsequently, the immunoprecipitated DNA was purified by phenol-chloroform extraction followed by ethanol precipitation. Thus obtained purified DNA was amplified by real time PCR using Universal SYBER green mix (Biorad) with indicated primers listed in Table S1(see Additional file 1). The data obtained was normalized with input DNA and expressed as fold DNA enrichment over pre immune sera.

### Flow cytometry analysis and cell viability assay

Flow cytometry of cells was done as described elsewhere [26]. Cell viability was examined in transfected cells using MTT assay. Cells were seeded at 0.5x10^6^ cells and transfected with vector or UBF expressing plasmid. After 48 h of transfection, cells were incubated with MTT at 37°C for 45 min. Crystals were solubilized using molecular grade DMSO and the absorbance was recorded at 560 nm using spectrophotometer. The mean absorbance values of three independent experiments were expressed as percentage of viability in relative to control cells.

### Immunofluorescence assay

Immunofluorescence assay was performed as described previously [28]. Briefly, Huh7 cells were transiently transfected with indicated expression plasmid. After 48 h post-transfection, cells were fixed with 2% formaldehyde and immunofluorescence staining was performed. The images were captured with the help of Nikon ECLIPSE TE 2000-U fluorescent microscope (Nikon Instrument Inc., USA) using 60x objective lens.

### Animal tumour model

The development of the X15-*myc* transgenic mice model of hepatocellular carcinoma (HCC) used in current study, has been described earlier [19]. PCR based methods were used to identify transgene-positive transgenic mice. Liver tissue samples were collected from different aged mice for the extraction of total RNA and protein samples. Normal mice of the same age groups served as internal control.

### Immunohistochemistry (IHC) of liver tissues

The avidin–biotin complex (ABC) method of IHC was used to determine UBF expression in the liver samples of normal C57/Blk6 and X15-*myc* transgenic mice by IHC. Briefly, paraffin sections of liver tissues were processed for antigen retrieval by first dewaxing in xylene, rehydration and treatment with 3% hydrogen peroxide followed by incubating in citrate buffer (pH 6.0) in a boiling water bath for 15–20 min. The sections were blocked with goat serum for 20 min and incubated with mouse anti-UBF (at a 1:300 dilution in PBS and 1% BSA). The samples were then processed for IHC analysis using the Dao Cytomation-LSAB system and an HRP kit (Dako) according to the manufacturer’s protocol. Finally, the sections were counterstained with haematoxylin and mounted with DPX. The bright field images were captured with the help of Nikon ECLIPSE TE 2000-U fluorescent microscope (Nikon Instrument Inc., Melville, NY, USA) using 20x objective lens.

### BrdU incorporation assay

Huh7 cells were seeded at a density of 0.1 million cells in a 12 well plate. They were transiently transfected with vector, HBx or UBF expression plasmid. After 48 hours post-transfection, cells were treated with thymidine (2 mM) for 24 hours. After 5 hour of release in complete media, cells were labelled with BrdU as per manufacturer’s guidelines (BrdU labelling kit, Roche Diagnostics). A total of 6 fields was selected to count the number of BrdU-positive cells and the total number of cells and the percentage of BrdU-positive cells was then estimated. The mean value of three experiments is presented.

### Cell transformation assay

IHH cells in a 6- well plate were transfected with with vector, HBx or UBF expression plasmids. After 72 h post-transfection, cells were trypsinized, and mixed with 0.4% agar in 2 × DMEM. The mixture was then layered upon 0.8% agar prepared in 2 × DMEM containing 10% FBS. Bright-field images of transformed colonies were captured with a Nikon ECLIPSE TE 2000-S microscope. An approximate of 10 random fields were selected to calculate the number of foci formed on each plate. The mean value of three experiments is presented.

### Statistical analysis

Statistical significance of results was calculated using Student’s t test. p value < 0.05 was considered as significant.

## Additional file

Additional file 1: Table S1. Oligonucleotide primers used for RT-qPCR and ChIP-qPCR. **Figure S1.** Expression levels of UBF in X15-*myc* transgenic mice. Immunohistochemical detection of UBF in the liver tissues of 6-month old control and X15-*myc* transgenic mice. (Original image magnification,×200). Scale bar represents10 um.

## Abbreviations

BrdU: Bromodeoxyuridine
ChIP: Chromatin immune-precipitation
HBV: Hepatitis B virus
HBx: X protein of HBV
HCC: Hepatocellular carcinoma
IHH: Immortalized human hepatocyte
RT-qPCR: Real-time quantitative PCR
UBF: Upstream binding factor
