# Supplementary material for: The HBx oncoprotein of hepatitis B virus potentiates cell transformation by inducing c-Myc-dependent expression of the RNA polymerase I transcription factor UBF
Source: Virol J. 2015 Apr 14;12:62. doi: 10.1186/s12985-015-0293-5 (PMC4424551; doi:10.1186/s12985-015-0293-5)
Supplement: Additional file 1: Table S1. — Oligonucleotide primers used for RT-qPCR and ChIP-qPCR. Figure S1. Expression levels of UBF in X15-myc transgenic mice. Immunohistochemical detection of UBF in the liver tissues of 6-month old control and X15-myc transgenic mice. (Original image magnification,×200). Scale bar represents10 um. [file 12985_2015_293_MOESM1_ESM.pdf]

Supplementary data

Primers	Sequence
ARPP P0 gene	F- 5' GCACTGGAAGTCCAACTACTTC 3'<br>R- 5' TGAGGTCCTCCTTGGTGAACAC3'
UBF gene	F- 5' AATTCAGGGAGAACCCAAG 3'<br>R- 5' TTAACCCAGAGGTCCAGGTG 3'
UBF promoter (ChIP primers)	F- 5' CACCCACTGATGAAGTCTATGAG 3'<br>R- 5' ACCCTTTTGAAGAGACACCAA 3'

Table S1. Oligonucleotide primers used for RT-qPCR and ChIP-qPCR.

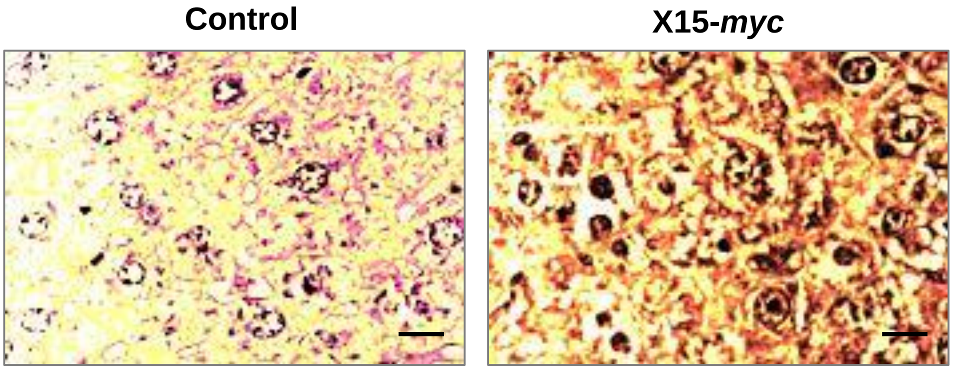

Figure S1. Expression levels of UBF in X15- *myc* transgenic mice. Immunohistochemical detection of UBF in the liver tissues of 6- month old control and X15-myc transgenic mice. (Original image magnification, × 200). Scale represents 10 μm.
